# Shear induced diffusion of platelets revisited

**DOI:** 10.3389/fphys.2022.985905

**Published:** 2022-10-13

**Authors:** Christos Kotsalos, Franck Raynaud, Jonas Lätt, Ritabrata Dutta, Frank Dubois, Karim Zouaoui Boudjeltia, Bastien Chopard

**Affiliations:** ^1^ Computer Science Department, University of Geneva, Geneva, Switzerland; ^2^ Department of Statistics, University of Warwick, Warwick, United Kindom; ^3^ Microgravity Research Center, Ecole Polytechnique, Université Libre de Bruxelles, Bruxelles, Belgium; ^4^ Laboratory of Experimental Medicine (ULB222), Faculty of Medicine, Université Libre de Bruxelles & CHU-Charleroi, Charleroi, Belgium

**Keywords:** platelets transport, platelets diffusion coefficient, shear induced diffusion, high-fidelity blood simulation, lattice Boltzmann method, high performance computing

## Abstract

The transport of platelets in blood is commonly assumed to obey an advection-diffusion equation with a diffusion constant given by the so-called Zydney-Colton theory. Here we reconsider this hypothesis based on experimental observations and numerical simulations including a fully resolved suspension of red blood cells and platelets subject to a shear. We observe that the transport of platelets perpendicular to the flow can be characterized by a non-trivial distribution of velocities with and exponential decreasing bulk, followed by a power law tail. We conclude that such distribution of velocities leads to diffusion of platelets about two orders of magnitude higher than predicted by Zydney-Colton theory. We tested this distribution with a minimal stochastic model of platelets deposition to cover space and time scales similar to our experimental results, and confirm that the exponential-powerlaw distribution of velocities results in a coefficient of diffusion significantly larger than predicted by the Zydney-Colton theory.

## 1 Introduction

Platelets, or thrombocytes, are an essential blood constituent, from a physiological and heamodynamical point of view. Their motion is mainly a consequence of mechanical and hydrodynamic interactions with deformable red blood cells and the plasma, which makes an accurate description of their transport challenging.

Among the blood constituents, platelets are the second most numerous cells in blood, after red blood cells, with a concentration of 150–450 × 10^9^/*L*. They are involved in multiple physiological and pathophysiological processes such as haemostasis, thrombosis, clot retraction, vessel constriction and repair, inflammation, including promotion of atherosclerosis, host defense, and even tumor growth/metastasis (Harrison (2005)). When needed, platelets respond rapidly through activation, adhesion, aggregation, release of the materials stored in their granules, *etc.* Any disorder in these physiological processes results in impaired haemostasis and inappropriate thrombus formation. For example, arterial thrombi can develop within atherosclerotic lesions resulting in stroke and heart attack, two of the major causes of morbidity and mortality in the western world Harrison (2005).

From a physical point of view, platelets are small rigid suspensions interacting with other, larger, deformable suspensions, the red blood cells. It is well recognized that the physics of such systems is rich and complicated Eckstein and Belgacem (1991); Kumar and Graham (2012). Processes such as margination and segregation are observed in which platelets move towards the wall of the system, while red blood cells concentrate in the center of the vessel, producing the so-called *cell free layer* at the wall (Kim et al. (2009); Fedosov et al. (2010); Carboni et al. (2016)).

Adhesion and aggregation of platelets depend not only on their affinity with the endothelium or the deposition surface but also on their flow towards this surface. Platelets movement in the blood is affected by their interactions with red blood cells (D’Apolito et al. (2015)). In order to properly interpret platelet function tests and evaluate if platelets are dysfunctional in a patient, it is important to identify and separate the effect of transport properties to the wall from the intrinsic biochemical platelets properties (adhesion and aggregation). In several diseases, the shape of red blood cells is modified, affecting platelet motion Boudjeltia et al. (2020). When analyzing the way platelets deposit on a surface, their adhesion and aggregation rates can only be determined if their flow is correctly known.

In a blood flow subject to a shear rate, platelets experience an enhanced random motion in the direction perpendicular to the flow. The accepted description of this process, the so-called Zydney-Colton theory (Zydney and Colton (1988)), is that platelets are subject to a diffusion process, whose diffusion coefficient depends on the shear rate $\dot{\gamma }$ and the hematocrit *H*, the fraction of space occupied by red blood cells Affeld et al. (2013):

$$D_{ZC}=D_{\mathrm{PRP}}\;\left(1-H\right)\;+\;0.15\;d_{\mathrm{RBC}}^{2}\;\left(H/4\right)\;\dot{\gamma }\;{\left(1-H\right)}^{1.8}$$ (1)

where *D*_*PRP*_ is the diffusivity of platelets in a platelet-rich plasma (i.e. without red blood cells) with a typical value of $D_{\mathrm{PRP}}=O\left(10^{-13}\right)\;m^{2}s^{-1}$ and *d*_*RBC*_ is the typical diameter of a red blood cell. For *H* = 0.35 and $\dot{\gamma }=100s^{-1}$ the value of *D*_*ZC*_ is

$$D_{ZC}=5\times 10^{-11}m^{2}s^{-1},$$ (2)

The present study was motivated by the determination of platelets adhesion and aggregation rates from *in-vitro* experiments with the Impact-R platelet analyzer device. It is made of a cylinder filled with whole blood and closed by two disks. The upper disk rotates to produce a prescribed shear flow and the lower disk is a deposition surface on which platelets can adhere and aggregate. In Chopard et al. (2017); Dutta et al. (2018, 2022) we show that platelet adhesion and aggregation rates can be computed from the *in-vitro* observations (deposition patterns in the Impact-R), by combining a mathematical model of platelets deposition and a machine learning technique. However, to explain the deposition pattern observed in our experiments, platelets must experience a large flow towards the deposition surface. More precisely, we found that with *H* = 0.35 and $\dot{\gamma }=100s^{-1}$ , a diffusion coefficient of the order 10^–8^ *m* ^2^ *s* ^−1^ is needed. This value is more than two orders of magnitude larger than the value predicted by Eq. 2.

The Zydney-Colton model has been extensively validated by numerous numerical studies in which red blood cells and platelets were resolved (Zhao and Shaqfeh (2011); Zhao et al. (2012); Reasor et al. (2013); Vahidkhah et al. (2014); Mehrabadi et al. (2015, 2016); Závodszky et al. (2019)). However, divergence from Zydney-Colton has also been observed and attributed to the presence of a drift term (Eckstein and Belgacem (1991); Crowl and Fogelson (2011); Kumar and Graham (2012)). While this leads to much higher diffusion than Zydney-Colton, it remains challenging to understand why such symmetry breaking occurs, at least with the geometry of the Impact-R.

In this context, we have developed a high-fidelity numerical blood flow solver, Palabos-npFEM, described and validated in Kotsalos et al. (2019, 2020). This computational framework is based on a lattice Boltzmann fluid solver with suspensions (red blood cells and platelets), whose deformable membranes are described with the finite element method (FEM). The fluid-blood cell interaction is computed with the immersed boundary method. Although not the purpose of the present discussion, we emphasize the fact that such numerical simulations are extremely demanding in computing resources, even for relatively small system sizes (much less than a cubic millimeter) and short periods (of the order of a second). In the present paper, we revisit platelets transport and propose a new characterization based on a probability distribution of their velocity. We estimate this distribution from high-fidelity blood flow simulations resolved at typical length and time scales similar to those considered in Müller et al. (2016); Závodszky et al. (2019), and we extrapolate platelets trajectory at much greater spatiotemporal scales using a surrogate stochastic model of platelets motion. We find that the resulting random walk process leads to a diffusion coefficient whose magnitude tends towards our *in-vitro* observations. We also argue that the traditional ways to compute the diffusion coefficient from the particle mean square displacement is very sensitive to finite size effects, hence resulting, for small systems, in a significant underestimation of the diffusion coefficient and questioning limitations of the Zydney-Colton theory at large spatiotemporal scales.

## 2 Methods

### 2.1 Palabos-npFEM framework

Palabos-npFEM is a computational framework for the simulation of blood flow with fully resolved constituents. The software computes the movement and deformation of red blood cells and platelets, and the complex interaction between them. The tool combines the lattice Boltzmann solver Palabos for the simulation of blood plasma (fluid phase), a finite element method (FEM) solver for the resolution of blood cells (solid phase), and an immersed boundary method (IBM) for the coupling of the two phases. Palabos-npFEM provides, on top of a CPU-only version, the option to simulate the deformable bodies on Graphic Processing Units (GPUs), thus the code is tailored for the fastest supercomputers Kotsalos et al. (2021).

In more details, the framework resolves blood cells like red blood cells and platelets individually (both trajectories and deformed state), including their detailed non-linear viscoelastic behavior and the complex interaction between them.

The fluid solver is based on the lattice Boltzmann method (LBM) and solves indirectly the weakly compressible Navier-Stokes equations. The solid solver is based on the nodal projective finite elements method (npFEM) Kotsalos et al. (2019), which offers an alternative way of describing elasticity. The npFEM framework is a mass-lumped linear finite element solver that resolves both the trajectories and deformations of the blood cells with high accuracy. The solver has the capability of capturing the rich and non-linear viscoelastic behaviour of red blood cells as shown and validated in Kotsalos et al. (2019). The platelets are simulated as nearly-rigid bodies by modifying the stiffness of the material. The implicit nature of the npFEM solver renders it capable of resolving extreme deformations with unconditional stability for arbitrary time steps. The fluid-solid interaction is realized by the immersed boundary method (IBM) and more specifically by the multi-direct forcing scheme proposed in Ota et al. (2012). The IBM imposes a no-slip boundary condition, so that each point of the surface and the ambient fluid moves with the same velocity.

Collisions between blood particles, whether red blood cells or platelets, are implemented through a repulsive force acting as a spring, when the surfaces delimiting two particles are getting too close to each other. In the current study, we employ the same parameters as reported in Kotsalos et al. (2019, 2020).

### 2.2 Probability distribution function of platelets absolute velocities

From the fully resolved simulations we have recorded the position *y*_*i*_(*t*), perpendicular to the flow, of the *i*th platelet, at every time interval *δt* = 10^–5^ *s*. We define the absolute velocity as:

$$v_{i}\left(t\right)=\frac{\left|y_{i}\left(t+\delta t\right)-y_{i}\left(t\right)\right|}{\delta t}$$ (3)

and computed the mean absolute velocity *v*_*moy*_:

$$v_{moy}=\frac{1}{N}\overset{N}{\underset{i=i}{\sum }}\left(\frac{1}{n}\overset{\left(n-1\right)\delta t}{\underset{t=0}{\sum }}v_{i}\left(t\right)\right)$$ (4)

where *N* is the total number of platelets and *n* the number of iterations.

We find that the probability distribution function of the platelets absolute velocities *P*(*v*) is well described by the relation:

$$P\left(v\right)=\left\{\begin{array}{cc} p_{0}\exp\left(-\lambda v\right) & \text{for}\;v\leq v_{min}\\ p_{0}\exp\left(-\lambda v_{\min}\right){\left(\frac{v}{v_{\min}}\right)}^{-1-\alpha } & \text{for}\;v\geq v_{min} \end{array}\right.$$ (5)

where *p*_0_, *λ*, *v*_min_ and *α* are parameters to be determined. The quantity *v*_min_, a velocity threshold separating the exponentially decreasing bulk of the distribution to its heavy tail, and *α* were estimated with the python package powerlaw Alstott et al. (2014), which is the python implementation of the seminal work of Clauset et al. (2009). Note that there has to be a lower bound as power-law distributions diverge when the variable tends to zero. More generally, power laws are observed in the tail, and these distributions deviate from the power law exhibiting various behaviors below some threshold value of the measured variable Redner (1998); Ioannides and Skouras (2013). We obtain the following values:

$$\alpha =3.8\;v_{min}=5\times 10^{-3}ms^{-1}\;v_{moy}=10^{-3}ms^{-1}$$ (6)

The values of *p*_0_ and *λ* are obtained from the conditions

$$1=\int_{0}^{\infty }dvP\left(v\right)=p_{0}\exp\left(-\lambda v_{min}\right)\left[\frac{v_{min}}{\alpha }-\frac{1}{\lambda }+\frac{p_{0}}{\lambda }\right]$$ (7)

and

$$v_{moy}=\int_{0}^{\infty }dv\;vP\left(v\right)=p_{0}\exp\left(-\lambda v_{min}\right)\left[\frac{v_{min}^{2}}{\alpha -1}-\frac{v_{min}}{\lambda }-\frac{1}{\lambda^{2}}\right]$$ (8)

These two equations for *p*_0_ and *λ* can be solved numerically, knowing *α*, *v*_min_ and *v*_*moy*_. The solution is *p*_0_ = 1015.24 and *λ* = 1017.36.

Here we assume that *v* can be arbitrarily large. This is of course not true in practice but adding to *P*(*v*) the numerically observed cut-off speed does not change our results because the probability of such high velocity decreases fast enough for the *α* value we have here. Similarly, imposing a low velocity threshold did not modify our results.

### 2.3 Stochastic model for platelets velocities

From the expression of *P*(*v*), one can sample the distribution and generate platelets velocities that mimic the behavior of the fully resolved blood flow simulation. We call this velocity sampling a stochastic model for platelets velocities. Concretely if *r* is a random number uniformly distributed in [0, 1], the value of *v* is sampled through the relation:

$$r=\int_{0}^{v}dv^{\prime }\;P\left(v^{\prime }\right)$$ (9)

The resulting velocity is:

$$v=\left\{\begin{array}{cc} -\frac{1}{\lambda }\ln\left(1-\frac{\lambda r}{p_{0}}\right) & \text{if}\;r<\frac{p_{0}}{\lambda }\left(1-\exp\left(-\lambda v_{min}\right)\right)\\ v_{min}{\left[1-\frac{\alpha }{p_{0}\exp\left(-\lambda v_{min}\right)}\left(r-\frac{p_{0}}{\lambda }\left(1-\exp\left(-\lambda v_{min}\right)\right)\right)\right]}^{-1/\alpha } & \text{if}\;r>\frac{p_{0}}{\lambda }\left(1-\exp\left(-\lambda v_{min}\right)\right) \end{array}\right.$$ (10)

Of course, when generating a velocity from *P*(*v*) we should remember that here, *v* is the absolute velocity, without its sign. The data from the simulation show that both signs of the platelets velocities are equally likely. This is expected due to the symmetry of the problem with respect to the *y*-axis. Therefore the sign of *v* is chosen at random with a probability 1/2 for both directions, independently of the value *v*. This is obtained by generating a second random number *r*′.

For each stochastic simulation we used the following procedure. First, we set the initial positions *y*_*i*_ (0) by placing randomly N platelets on a segment of length L. Then at each iteration the velocities *v*_*i*_ were drawn from the distribution described previously and the platelets positions were updated accordingly 3. A platelet *i* with a position *y*_*i*_ ≤ 0 is removed from the system (deposited platelet), while the boundary *y*_*i*_ > *L* is reflective. Based on velocity auto-correlation measurements discussed below, we set *δt* = 0.5 *ms*.

## 3 Results and discussion

**FIGURE 1:**
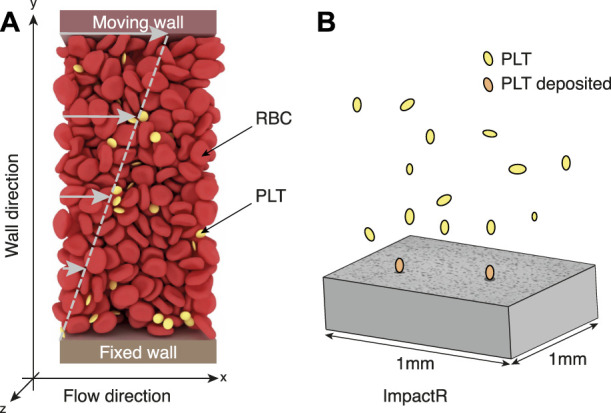
**(A)** A typical configuration of our high-fidelity blood simulation, with deformable red blood cells and platelets (yellow particles) in suspension in a Newtonian flow subject to a shear rate. In this setup, fluid velocity increases linearly from 0 (at the bottom fixed wall) to *V*_*mw*_ (velocity of the top moving wall). The system has periodic boundary conditions along the *x* and *z* axis, while the *y*-direction (or wall direction) is bounded by walls. **(B)** Illustration of the process taking place in the Impact-R platelets analyzer. Under the action of a shear flow and their interaction with red blood cells, platelets move towards the bottom of the system where they deposit, forming aggregates displayed on the gray slab as dark clusters.

We have performed simulations of the system depicted in Figure 1A, for shear rates 100 s^−1^ and an hematocrit of 35% as in the Impact-R experiments. We have analyzed platelets trajectories along the vertical direction, that is perpendicular to the flow. We consider these simulations for 1*s* of physical time and for a system of size 50*μm* × 50 *μm* horizontally, and of height *L*, with *L* ∈ {50, 100, 250, 500} *μm* in the vertical direction. Periodic boundary conditions are imposed horizontally along the *x* and *z* directions, while the *y* direction (or wall direction) is bounded by walls. The upper moving wall has a constant velocity *V*_*mw*_ so as to produce the desired shear flow. Although these simulations are significantly smaller in size and time than the actual Impact-R experiment, they require supercomputing capabilities.

In what follows, we focus on shear rate 100*s* ^−1^ as in the Impact-R experiments, and on the smallest system of size *L* = 50 *μm*. The reason is two-fold. First this is the typical size that has been already considered in the literature for high-fidelity blood flow numerical simulation. Second, due to the small size, the flow reaches much faster a steady state on which reliable measurements can be considered.

**FIGURE 2:**
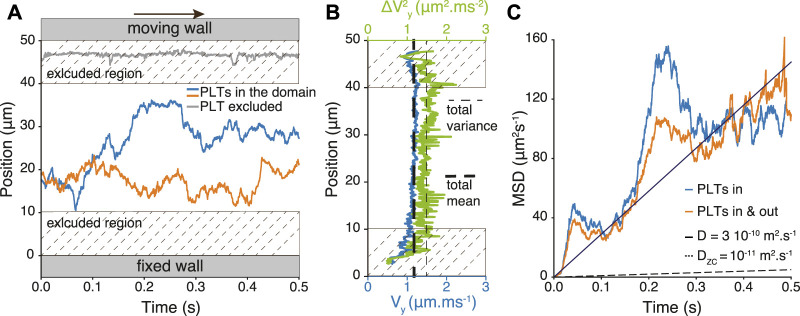
**(A)** Sketch of the system with the region in which platelets trajectories are recorded. Trajectories *y*(*t*) of two representative platelets along the direction perpendicular to the flow. Platelets (gray) with trajectory inside the excluded region are not considered for analysis. The situation shown in this figure corresponds to a wall to wall distance of *L* = 50*μm*, hematocrit *H* = 0.35 and shear rate $\dot{\gamma }=100s^{-1}$ . **(B)** Profiles of the velocity *V*_*y*_ (blue) and the variance of the velocity $\Delta V_{y}^{2}$ (green) along the wall direction y. Velocity and its variance are computed in bins of 0.1 *μm* and bins with frequency lower than 0.1% are not represented. The total mean (resp. variance) in thick (resp. thin) dashed line is computed away from the wall (between *y* = 10 *μm* and *y* = 40 *μm*). **(C)** The mean square displacement MSD(*t*) measured within the system limits is shown, as well as the corresponding diffusion coefficient. The blue curve represents the MSD measured from the platelets that remain in the domain of interest during all the simulation time (in), while orange curve is computed from the trajectories of the platelets during their residency in the domain (in and out). The dashed straight line represents the MSD expected by Zydney-Colton theory.

A typical trajectory is shown in Figure 2A. Note that we consider the trajectory as long as the platelet stays far enough from the boundaries. Indeed, we observe in our high-fidelity simulations that the trajectory of the platelets is strongly affected by the presence of the walls (see Figure 2A). This is the so-called margination process that naturally brings and traps platelets close to walls. To obtain data not affected by the system boundaries, the region we consider goes from *y* = 10 *μm* to *y* = *L* − 10*μm*, which corresponds to discarding from the analysis a bottom and a top layer of about one red blood cell diameter. Note also that, due to the symmetry of the flow, the density of red blood cells is uniform along *y* so that the vertical platelets transport is independent of *y* as shown by the profiles of the velocity and its variance along the wall direction (Figure 2B). Such symmetry consideration does not hold anymore in different geometries, for instance in a tube, where the shear rate is not uniform.

From the values of *y*_*i*_(*t*), it is traditional to compute the platelets mean square displacement MSD(*t*) as

$$MSD\left(t\right)={\left\langle {\left[y_{i}\left(t\right)-y_{i}\left(0\right)\right]}^{2}\right\rangle }_{i}$$ (11)

where ⟨⋅⟩_*i*_ indicates an average over the platelets, in our case those that are still in the domain at time *t*. The diffusion coefficient is linked to the means square displacement, as *D*_*MSD*_ = MSD(*t*)/(2*t*) for 1D systems (Figure 2B).

We consider platelets that remain in the domain of interest during all the simulation time to compute a first quantity *D*_*in*_. Further, we compute *D*_*in*&*out*_ from the trajectories of all the platelets until they leave the domain.

**FIGURE 3:**
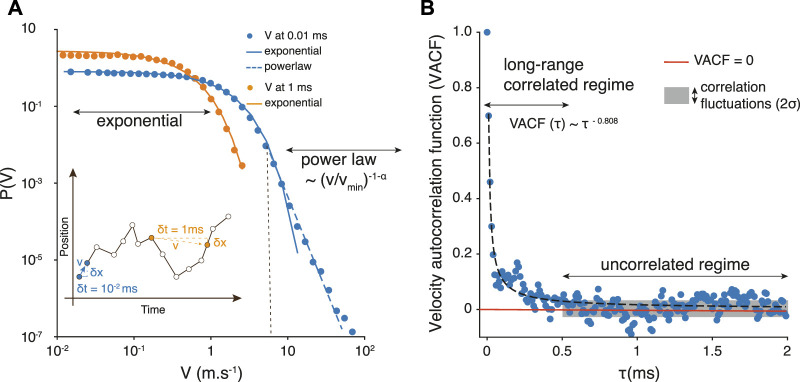
**(A)** Velocity probability distribution *P*(*v*) obtained from sampling the platelets trajectories at a time resolution *δt* = 10^–5^ *s* (blue), and *δt* = 10^–3^ *s* (orange). The straight lines represent an exponential decrease and the dashed one a powerlaw behavior, hence suggesting a velocity distribution described by Eq. 5. The distributions were computed with logarithmically spaced bins. The inset indicates how velocities are measured from trajectories samples at different values of *δt*. **(B)** Platelet velocity auto-correlation function $VACF\left(\tau \right)={\left\langle v_{i}\left(t+\tau \right)v_{i}\left(t\right)\right\rangle }_{i}$ as a function of the time delay *τ*. The fit of the data points gives a power law relation, VACF(*τ*) ∼ *τ* ^−*b*^, with *b* = 0.808.

We obtain, for the present small system, *D*_*in*&*out*_ = 1.71 × 10^−10^ *m* ^2^ *s* ^−1^ and *D*_*in*_ = 1.99 × 10^−10^ *m* ^2^ *s* ^−1^ (Figure 2), which is compatible with previous numerical observations (see for instance Reasor et al. (2013)), but larger than the MSD(*t*) that would emerge from a diffusion constant given by *D*_*ZC*_. However, as shown below, the determination of *D* directly from the platelets trajectories in such small spatial system is not accurate and we claim that it underestimates *D*. Therefore we will consider below a different procedure, relating the diffusion coefficient to the velocity probability distribution (Figure 3A and Method) and velocity auto-correlation function.

Our goal with this stochastic model is to generate representative platelets trajectories, much faster than with the fully resolved blood flow simulation, and in any geometry, including scales and time span similar to the Impact-R experiment. To do so, we still need to determine the mean time between two random changes of *v*. This is obtained by measuring the velocity auto-correlation function ${\left\langle v_{i}\left(t\right)v_{i}\left(t+\tau \right)\right\rangle }_{i}$ as a function of *τ*. The results obtained from the high-fidelity blood simulation are shown in Figure 3B. We observe that

$$VACF\left(\tau \right)=\left\langle v\left(t+\tau \right)v\left(t\right)\right\rangle =a\tau^{-b}$$ (12)

with *a* = 0.017 × 10^−3^ *s* (SD = 0.0015) and *b* = 0.808 (SD = 0.029), when *τ* is expressed in *ms*.

The fact that VACF(*t*) is not an exponential function indicates that platelets keep a memory of their velocity over a rather large time interval Δ*t*. The effect of these long-range correlations is significant when sampling the trajectories at different rates. For *δt* = 10^−3^ *s*, the velocity histogram captures only the exponential part of the distribution, suggesting that the tail of the distribution results from short-time correlated platelets displacements (see the inset in Figure 3A). To determine the value of Δ*t* we compute the standard deviation *σ* of the distribution of values in the tail of VACF(*τ*) (gray box in Figure 3B). The value of *τ* above which measurement points match the value of *σ* is considered as the typical time Δ*t* after which the velocity is randomized. Therefore we define

$$\Delta t=0.5\times 10^{-3}s$$ (13)

From the value of Δ*t* and the distribution *P*(*v*), we can use the stochastic model to generate representative platelets trajectories at much larger spatial and temporal scales, since it requires very modest computational resources.

A typical trajectory is obtained as

$$y\left(t+\Delta t\right)=y\left(t\right)+v\left(t\right)\Delta t$$ (14)

with

$$v\left(t\right)∼\pm P\left(v\right)$$ (15)

where the notation ∼±*P*(*v*) means that a new velocity is drawn from *P*(*v*) with an equally likely positive or negative sign.

**FIGURE 4:**
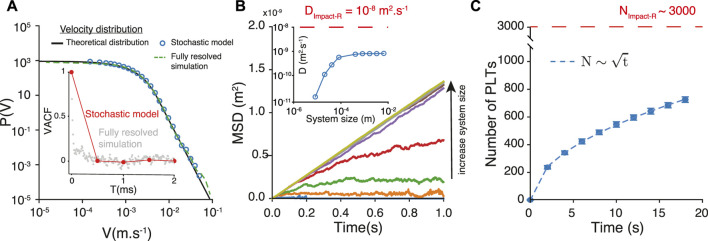
**(A)** Velocity probability distribution obtained from the fully resolved simulation, the analytical expression and the stochastic model. **(B)** Mean square displacement obtained with the stochastic model of trajectories, for different system sizes. The inset show of the corresponding diffusion coefficient is sensitive to the size of the system, for small systems. The red dashed line represents the diffusion coefficient estimated from the Impact-R experiments. **(C)** The number N of platelets that deposit as a function of time for a system of size *L* = 0.82 *mm* and initially 4,800 platelets uniformly distributed along *L*. The red dashed line represented the number of deposited platelets measured on the Impact-R device.

Figure 4A compares the velocity distributions of the stochastic model, the high-fidelity model and the analytical expression. We measure a Pearson correlation coefficient 0.999 (*p*_*val*_ < 10^–64^) between the analytical expression and the distribution of velocity of the stochastic model, and a correlation coefficient 0.995 (*p*_*val*_ < 10^–35^) between the analytical expression and the distribution of velocity of the fully resolved model.

Figure 4B shows the mean square displacement MSD(*t*) corresponding to particles following this stochastic model. We investigate the finite size effects on the measure of the MSD(*t*) and the estimation of the diffusion coefficient *D*. For small system sizes, the MSD saturates, hence resulting in spurious estimation of the diffusion coefficient (inset Figure 4B). Note that the asymptotic value of D can only be found at large system sizes. Increasing only the simulation time is not sufficient as the saturation of the MSD is still present after 20*s*. For the largest system, 10^−2^ *m*, the diffusion coefficient associated with MSD(*t*) is:

$$D=0.67810^{-9}m^{2}s^{-1}$$ (16)

which is more than 65 times larger than *D*_*zc*_. It is also about 4 times larger than the value obtained from a direct measurement of MSD(*t*) as shown in Figure 4B.

One can further use our stochastic model to simulate the number N of platelets that deposit within 20 s in the Impact-R. In the simulation, platelets whose trajectory reaches the deposition surface at *y* = 0 are counted as absorbed and removed from the system. Those reaching the upper boundary at *y* = *L* = 0.82 *mm* are bounced back. This process can be iterated for 20*s* with an initial number of 4,800 platelets uniformly distributed along *L*. The total number of platelets that reach the absorbing boundary *y* = 0 increases in time as $\sqrt{t}$ , as predicted from the survival probability in a 1*D* diffusion-absorption process. After 20*s* we measure near 800 deposited platelets (see Figure 4C). This is however still significantly less than the 3,000 platelets observed to deposit in the Impact-R during this same time interval.

**FIGURE 5:**
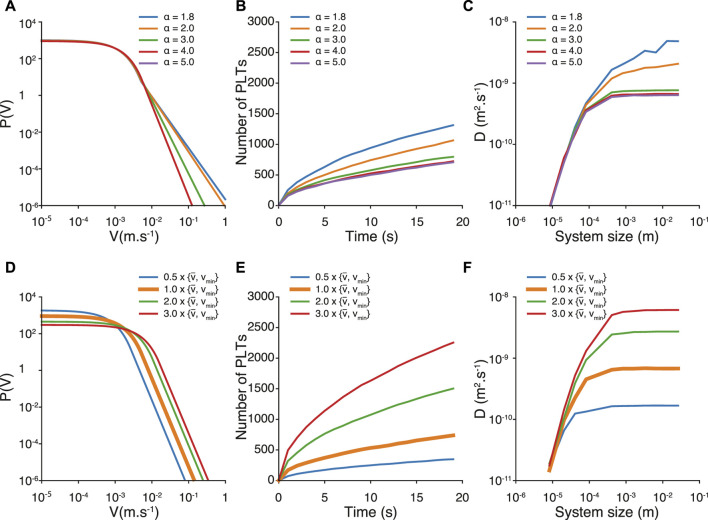
Velocity distribution, number of deposited platelets and finite size effect of the diffusion coefficient for different values of *α* **(A–C)** and different values of *v*_*moy*_ and *v*_min_ **(D–F)**. In Figures **(D–F)**, the orange curve represents our reference values of *v*_*moy*_ and *v*_min_.

It is important to notice that our results are sensitive to the values *α*, *v*_min_ and *v*_*moy*_ measured from the velocity distribution given in Figure 3A. We first investigate the effect of the parameter*α* in Figures 5A–C. Changing the value of *α* only modifies the tail of the distribution, whereas the exponentially decreasing bulk of the distribution remains unchanged (see Figure 5A). We explored the set of following values *α* = 1.8, 2, 3, 4, and 5. This set of values includes the very peculiar Levy-flight regime that is characterized by infinite variance Viswanathan et al. (2008). We do not observe significant change, both for the number of platelets and the diffusion coefficient, for values of *α* above 2 (Figures 5B,C). Instead, the diffusion coefficient *D* increases as *α* decreases below 2, and we measure *D* = 4.4 10^−9^ *m* ^2^ *s* ^−1^ and approximately 1300 deposited platelets. Our fully resolved simulation does not show such values for *α*, but we cannot exclude totally the existence of such a regime for simulated systems with size and time scales comparable to those of the Impact-R setup. Yet speculative, these results show that except for the Levy-flight regime, the parameter *α* does not modify significantly the transport properties of the platelets.

We then investigate the effect of changing *v*_*moy*_ and *v*_min_. For a sake of simplicity, and also to keep the distribution of velocity smooth, we multiply concordantly *v*_*moy*_ and *v*_min_ by values between 0.5 and 3 (see Figure 5C). We were motivated to increase the mean velocity by the fact that the observed mean velocity in the fully resolved model is related to the actual time resolution of the model. Indeed, decreasing the time resolution results in an increase of the mean velocity. Typically, the mean velocity increases by a factor 2 when the time resolution is decreased by a factor 100. This implies that at the fluid resolution time *δt* = 10^−7^ *s*, one could expect a platelets mean velocity twice greater than the one measured at *δt* = 10^−5^ *s*. Simulations at lower time resolution can be envisioned for future work, however at a dramatic computational cost. According to the different velocity distributions (see Figure 5D), we observe that both the number of deposited platelets and diffusion coefficient increase with the values of *v*_*moy*_ and *v*_min_. Interestingly, for the highest *v*_*moy*_ we tested, we find a diffusion coefficient similar to the one obtained with *α* < 2 (*D* = 6.11 10^–9^ *m* ^2^ *s* ^−1^), but a number N of platelets twice greater. This suggests the predominant role of the platelets mean velocity to explain the number N observed in the Impact-R experiment.

## 4 Conclusion

This paper proposes a detailed analysis of the statistics of platelets velocities when subject to an imposed shear flow of red blood cells and plasma. Our motivation was to better understand the deviation between the transport properties and deposition of platelets predicted by the Zydney-Colton relation 1) and those inferred from our Impact-R experiments.

Using fully resolved blood flow numerical simulations, in which deformable red blood cells and platelets are in suspension in a shear rate flow created between two walls, we were able to reconstruct the probability distribution *P*(*v*) of the platelets velocities. Such simulations are extremely time consuming as they have to solve the flow, the deformability of red blood cells, and the interaction between the fluids and the blood cells, as well as the interaction between these blood cells. Due to this high computational requirement, our study is limited here to systems of size 50*μm* × 50*μm* × 50*μm*, for 1 s of physical time.

From the simulation data, we found that *P*(*v*) is made of an exponential part followed by a power law tail. We also determine the velocity auto-correlation function and its characteristic time scale Δ*t* for velocity decorrelation. From *P*(*v*) and Δ*t* one can propose a stochastic model to generate platelets trajectories that mimic actual trajectories. However, with this stochastic model one can consider much larger spatial and temporal scales. At such larger scales, we found that the platelets diffusion coefficient perpendicular to the flow direction is about 65 times larger than *D*_*ZC*_, predicted by the Zydney-Colton theory. However, for small system sizes, the diffusion coefficient inferred from the evaluation of the MSD within the systems boundaries gives a value of the same order than predicted by the ZC relation. More *in-silico* experiments, yet at high computational cost, are critical to extend our findings in a broader parameter space, including higher shear rate values, and validate our description in terms of probability of velocities distribution and diffusion coefficient at larger scales.

Overall our results go in the direction of the experimental observation about the flux of platelets in the Impact-R device, which requires a much larger diffusion coefficient than *D*_*ZC*_ but still a diffusion constant at least one order of magnitude larger than what is found here. More analysis is still needed to clarify this discrepancy.

In view of the importance of a right characterization of platelets transport in clinical devices to correctly test platelets functionality, we hope that this study will stimulate more experimental and numerical work.

## Data Availability

The datasets generated and analyzed for this study can be found on Zenodo: https://zenodo.org/record/7028165.
