# Erratum: Shear induced diffusion of platelets revisited

**DOI:** 10.3389/fphys.2023.1175413

**Published:** 2023-03-08

**Authors:** Frontiers Production Office

**Affiliations:** Frontiers Media SA, Lausanne, Switzerland

**Keywords:** platelets transport, platelets diffusion coefficient, shear induced diffusion, high-fidelity blood simulation, lattice Boltzmann method, high performance computing

An omission to the **Fundin** **g** section of the original article was made in error. The following sentence has been added: “Open access funding was provided by the University of Geneva.”

The original version of this article has been updated.

